# Prognostic Value of Clinical and Laboratory Markers in Hospitalized Influenza‐Positive Patients: A Retrospective Cohort Study With ROC‐Based Risk Stratification

**DOI:** 10.1002/hsr2.72991

**Published:** 2026-08-05

**Authors:** Mohsen Daneshmand, Arefeh Ebrahimian Shiadeh, Farzane Sadeghi, Hemmat Gholinia, Farzin Sadeghi, Masomeh Bayani

**Affiliations:** ^1^ Student Research Committee Babol University of Medical Sciences Babol Iran; ^2^ Department of Medical Microbiology, Faculty of Medicine Golestan University of Medical Science Gorgan Iran; ^3^ Department of Gynecology and Obstetrics, School of Medicine Babol University of Medical Sciences Babol Iran; ^4^ Social Determinants of Health Research Center, Health Research Institute Babol University of Medical Sciences Babol Iran; ^5^ Cellular and Molecular Biology Research Center, Health Research Institute Babol University of Medical Sciences Babol Iran; ^6^ Infectious Diseases and Tropical Medicine Research Center, Health Research Institute Babol University of Medical Sciences Babol Iran

**Keywords:** biomarkers, CRP, ICU, influenza, LDH, mortality, prognosis, risk stratification

## Abstract

**Background and Aims:**

Seasonal influenza continues to cause substantial morbidity and mortality worldwide. Identifying reliable clinical and paraclinical predictors of adverse outcomes may aid in early triage and management. This study aimed to evaluate clinical and paraclinical factors associated with prolonged hospitalization, ICU admission, and in‐hospital mortality in influenza‑positive patients.

**Methods:**

This retrospective study analyzed 294 RT‐PCR–confirmed influenza cases admitted to Ayatollah Rouhani Hospital (Babol, Iran) during fall–winter 2022–2023. Demographic, clinical, and laboratory data were extracted. Key outcomes were prolonged hospital stay (> 5 days), ICU admission, and in‐hospital death. Logistic regression and ROC analysis were used to identify independent predictors and cutoff values.

**Results:**

Prolonged hospitalization occurred in 43.5% of patients, ICU admission in 8.8%, and mortality in 5.0%. In multivariate analysis, opium use (OR: 5.4), renal disease (OR: 9.27), and LDH > 1000 (OR: 8.74) were significantly associated with longer hospital stay. LDH > 1000 also predicted ICU admission (OR: 46.9) and mortality (OR: 21.7). Oxygen saturation < 93% increased mortality risk (OR: 7.98). ROC analysis identified LDH ≥ 633 and CRP ≥ 88.5 as optimal cutoffs for predicting mortality.

**Conclusion:**

Readily available markers such as LDH, CRP, liver enzymes, and oxygen saturation, which showed significant associations with prolonged hospitalization, ICU admission, or mortality in our analyses, may assist in early risk stratification of hospitalized influenza patients.

## 1 Introduction

Influenza is an acute respiratory illness caused by influenza viruses, primarily types A and B, which are responsible for seasonal epidemics worldwide [1, 2]. Each year, influenza is associated with an estimated 290,000 to 650,000 deaths globally, posing a major public health concern, particularly during winter months [3]. These viruses belong to the Orthomyxoviridae family and are characterized by negative‐sense, single‐stranded RNA genomes [4]. Transmission occurs mainly through respiratory droplets, with additional spread via aerosols and contaminated surfaces [5, 6]. Following an incubation period of one to 4 days, influenza typically presents with sudden onset of fever, cough, sore throat, headache, myalgia, and malaise [7, 8]. While most infections are self‐limiting, severe cases, particularly in the elderly, pregnant women, and individuals with underlying comorbidities, can lead to pneumonia, respiratory failure, and death [9]. Clinical and laboratory parameters have been studied as potential indicators of disease severity. Common laboratory abnormalities in hospitalized patients include leukocytosis, lymphopenia, and elevated inflammatory markers such as C‐reactive protein (CRP) and lactate dehydrogenase (LDH) [10]. However, the predictive value of these laboratory parameters remains uncertain, and there is currently no validated risk stratification tool specifically designed for hospitalized influenza patients [11]. Moreover, existing studies often focus on isolated outcomes such as ICU admission or mortality, without integrating a broader spectrum of clinical prognostic endpoints [12]. Therefore, a comprehensive evaluation of both clinical and paraclinical indicators across multiple outcomes is needed to enhance early risk assessment and optimize care. This retrospective study aimed to evaluate the clinical and paraclinical predictors of adverse outcomes, including prolonged hospitalization, ICU admission, and in‐hospital mortality, among influenza‐positive patients admitted to Ayatollah Rouhani Hospital, Babol, Iran during the 2022–2023 influenza season.

## 2 Methodology

### 2.1 Study Design

This retrospective cohort study was conducted at Ayatollah Rouhani Hospital, a major referral center in Babol, northern Iran. The study included patients admitted between October 2022 and March 2023 (fall–winter influenza season), who were confirmed to be influenza‐positive by RT‐PCR. All hospitalized patients with laboratory‐confirmed influenza (via RT‐PCR) during the study period were screened. Of the initial 315 medical records from 309 patients, 294 cases met the inclusion criteria and were included in the final analysis. Sample size estimation was performed using PASS version 15 software, targeting an odds ratio of 2.19, a significance level (α) of 0.05, power (1–β) of 0.98, and accounting for 20% attrition, yielding a minimum required sample size of 285 patients.

### 2.2 Study Population and Sampling

The inclusion criteria comprised hospitalized patients with RT‐PCR–confirmed influenza who were either discharged by physician decision or died during hospitalization. Patients who had a hospital stay longer than 5 days or were admitted to the ICU were also included, even if they were discharged against medical advice. Exclusion criteria included patients who were discharged against medical advice within 5 days of admission, and those who developed symptoms after 48 h of admission, in order to exclude nosocomial infections.

### 2.3 Data Collection

Data were extracted from both paper‐based and electronic health records using a structured form. The following variables were collected:

#### 2.3.1 Demographic and Clinical Predictors

Age, sex, duration of symptoms before admission, presence of fever at admission, initial oxygen saturation (SpO_2_), and documented comorbidities (including cardiovascular disease, diabetes mellitus, chronic pulmonary disease, and renal disease). History of tobacco and opium use was also recorded. Fever was defined as a documented body temperature ≥ 38.0°C (100.4 °F) at the time of admission or recorded as ‘fever’ in the physician's clinical notes.

#### 2.3.2 Laboratory Predictors

Initial routine laboratory values prior to influenza diagnosis were obtained, including complete blood count (CBC) with differential, C‐reactive protein (CRP), erythrocyte sedimentation rate (ESR), lactate dehydrogenase (LDH), aspartate transaminase (AST), alanine transaminase (ALT), blood urea nitrogen (BUN), and creatinine.

#### 2.3.3 Definitions of Outcome Measures

Prolonged hospitalization was defined as a hospital stay longer than 5 days. ICU admission referred to any transfer to the intensive care unit during hospitalization. Mortality was defined as all‐cause in‐hospital death occurring during the same admission.

Comorbidities, including hypertension, were extracted from patients’ medical records based on documented physician diagnoses from the medical history, not solely on admission vital signs. Detailed data on blood pressure measurements, antihypertensive medication use, or baseline blood pressure control status were not systematically collected due to the retrospective design. All laboratory values reported represent initial measurements obtained at hospital admission (or within the first 24 h of admission). Pre‐influenza baseline laboratory data were not available for the majority of patients due to the retrospective design and lack of routinely collected outpatient laboratory records.

### 2.4 Statistical Analysis

All statistical analyses were performed and reported in accordance with the SAMPL (Statistical Analyses and Methods in the Published Literature) guidelines. Descriptive statistics were used to summarize baseline demographic, clinical, and laboratory characteristics. Categorical variables were reported as frequencies and percentages, and continuous variables as means ± standard deviation (SD) or medians with interquartile ranges (IQR), as appropriate. To evaluate associations between potential predictors and clinical outcomes (prolonged hospitalization, ICU admission, and in‐hospital mortality), univariate logistic regression analyses were first conducted. Variables with a *p*‐value < 0.20 in the univariate analysis were considered for inclusion in multivariate models. Multivariate stepwise logistic regression models were then constructed to identify independent predictors. Adjusted odds ratios (ORs) with 95% confidence intervals (CIs) were calculated. All statistical tests were two‑sided, and a *p*‐value < 0.05 was considered statistically significant. Receiver Operating Characteristic (ROC) curve analysis was performed to assess the discriminative performance of pre‐specified biomarkers (LDH and CRP). ROC analyses performed for additional biomarkers (ESR and NLR [neutrophil‐to‐lymphocyte ratio]) were considered exploratory. The area under the ROC curve (AUC) with 95% CI was calculated, and optimal cutoff points with corresponding sensitivity and specificity values were determined. In addition, to characterize the inter‑relationships among admission biomarkers, pairwise correlation analyses were performed. Pearson correlation coefficients were calculated for ESR, CRP, creatinine (Cr), blood urea nitrogen (BUN), AST, ALT, and LDH; for biomarkers with non‑normal distributions, Spearman rank correlation coefficients were additionally examined to confirm the robustness of the findings. Correlation matrices were generated to summarize the strength and direction of these associations. At the end of the Methods section, all statistical tests used in the analyses are summarized as follows: chi‐square test or Fisher's exact test for categorical variables; independent‐samples t‐test or Mann–Whitney U test for continuous variables; univariate and multivariate logistic regression models to estimate ORs and CIs; and ROC curve analysis with AUC estimation. All statistical analyses were conducted using SPSS software (SPSS version 24; IBM Corp., Armonk, NY, USA).

### 2.5 Data Quality Control

Data accuracy was ensured through double‐entry verification and independent review by two researchers. All patient identifiers were anonymized and deidentified prior to data analysis to ensure confidentiality and compliance with ethical standards.

### 2.6 Ethical Considerations

This study was approved by the Ethics Committee of Babol University of Medical Sciences (Approval ID: IR.MUBABOL.HRI.REC.1402.058). Owing to the retrospective design and the use of anonymized medical records, the requirement for written informed consent was waived by the ethics committee.

## 3 Results

### 3.1 Baseline Characteristics

A total of 294 hospitalized patients with laboratory‐confirmed influenza were enrolled in the study. The mean age was 58.97 ± 20.39 years, and 54.1% were female. Among female patients, 23.3% were pregnant. Fever was documented in 26.4% of patients, and 31.2% had oxygen saturation below 93%. Regarding comorbid conditions, hypertension (39.5%), cardiovascular disease (31.3%), and diabetes mellitus (27.0%) were the most common. Opium use was reported in 20.7% of patients. Paraclinical findings revealed leukocytosis in 18.0%, lymphopenia in 36.5%, and thrombocytopenia in 25.9% of cases. Elevated ESR and CRP were observed in over 60% of patients. Elevated AST and ALT levels were noted in 37.5% and 17.9% of patients, respectively. LDH levels > 1000 U/L were found in 6.7% of the cohort. Baseline demographic and clinical characteristics of the study population are summarized in Table 1.

**Table 1:** Baseline demographic and clinical characteristics of patients (*n* = 294).

Variable	Category	*n* (%)
Age	< 65 years	173 (58.8%)
	≥ 65 years	121 (41.2%)
Gender	Female	159 (54.1%)
	Male	135 (45.9%)
Symptom duration	≤ 3 days	139 (56.5%)
	> 3 days	107 (43.5%)
Fever	No	198 (73.6%)
	Yes	71 (26.4%)
Oxygen saturation	≥ 93%	174 (68.8%)
	< 93%	79 (31.2%)
Chronic pulmonary disease	No	208 (77.6%)
	Yes	60 (22.4%)
Cardiovascular disease	No	184 (68.7%)
	Yes	84 (31.3%)
Hypertension	No	161 (60.5%)
	Yes	105 (39.5%)
Kidney disease	No	244 (91.7%)
	Yes	22 (8.3%)
Liver disease	No	264 (98.9%)
	Yes	3 (1.1%)
Diabetes mellitus	No	195 (73.0%)
	Yes	72 (27.0%)
Thyroid dysfunction	No	234 (87.6%)
	Yes	33 (12.4%)
Active malignancy	No	251 (94.0%)
	Yes	16 (6.0%)
Rheumatologic disorders	No	253 (94.8%)
	Yes	14 (5.2%)
Neurological disorders	No	237 (88.8%)
	Yes	30 (11.2%)
Tobacco use	No	184 (80.7%)
	Yes	44 (19.3%)
Opium/opioid use	No	180 (79.3%)
	Yes	47 (20.7%)

*Note:* Data are presented as number (percentage). This table describes the characteristics of the study population; no statistical comparisons were performed.

### 3.2 Clinical Outcomes

Among the studied patients, 43.5% experienced prolonged hospitalization (> 5 days), 8.8% were admitted to the intensive care unit (ICU), and 5.0% died during hospitalization. The distribution of clinical outcomes, including length of hospital stay, ICU admission, and mortality, is detailed in Table 2.

**Table 2:** Clinical outcomes of hospitalized influenza patients (*n *= 294).

Outcome	Category	*n* (%)
Length of hospital stay	≤ 5 days	166 (56.5%)
	> 5 days	128 (43.5%)
ICU admission	Yes	25 (8.8%)
	No	258 (91.2%)
Mortality	Yes	14 (5.0%)
	No	266 (95.0%)

*Note:* Data are presented as number (percentage) of the total study population. No statistical analysis was conducted for this descriptive summary.

### 3.3 Paraclinical Findings

All paraclinical values reported in Table 3 represent initial measurements obtained at hospital admission. Pre‐influenza baseline laboratory data were not available for comparison; therefore, these abnormalities may reflect a combination of pre‐existing conditions and acute influenza‐related changes. Among the 294 patients included in the study, the most common paraclinical abnormalities were anemia (50.0%), leukocytosis (18.0%), leukopenia (9.2%), neutropenia (2.6%), and lymphopenia (36.5%). A neutrophil‐to‐lymphocyte ratio (NLR) < 2.5 was observed in 55.8% of cases. Thrombocytosis was rare (1.7%), whereas thrombocytopenia was detected in 25.9% of patients. With respect to inflammatory and biochemical markers, 5.6% of patients had an ESR > 100 mm/hr. Elevated creatinine (> 1.2 mg/dL) and BUN (> 20 mg/dL) were noted in 39.3% and 39.7% of patients, respectively. Elevated liver enzymes were also frequent, with AST > 40 IU/L in 37.5% and ALT > 40 IU/L in 17.9%. High LDH levels were common: 60.0% had LDH between 500 and 1000 U/L and 6.7% had LDH > 1000 U/L. Abnormal coagulation parameters included INR > 1.5 in 3.3% and PTT > 40 s in 9.3% of patients. CRP levels varied widely: 19.9% had CRP between 0 and 10 mg/L and 20.8% had levels > 100 mg/L. Full paraclinical characteristics are provided in Table 3.

**Table 3:** Paraclinical characteristics of the study population (*n* = 294).

Variable	Category	*n* (%)
Anemia	Yes	147 (50.0%)
	No	147 (50.0%)
Leukocytosis	Yes	53 (18.0%)
	No	241 (82.0%)
Leukopenia	Yes	27 (9.2%)
	No	267 (90.8%)
Neutropenia	Yes	5 (2.6%)
	No	188 (97.4%)
Lymphopenia	Yes	70 (36.5%)
	No	122 (63.5%)
NLR	< 2.5	164 (55.8%)
	≥ 2.5	130 (44.2%)
Thrombocytosis	Yes	5 (1.7%)
	No	289 (98.3%)
Thrombocytopenia	Yes	76 (25.9%)
	No	218 (74.1%)
ESR (mm/hr)	> 100	16 (5.6%)
	60–100	39 (13.7%)
	30–60	77 (27.0%)
	10–30	111 (38.9%)
	0–10	42 (14.7%)
CRP (mg/L)	> 100	45 (20.8%)
	60–100	40 (18.5%)
	30–60	39 (18.1%)
	10–30	49 (22.7%)
	0–10	43 (19.9%)
Creatinine	> 1.2	114 (39.3%)
	≤ 1.2	176 (60.7%)
BUN	> 20	114 (39.7%)
	≤ 20	173 (60.3%)
AST	> 40	84 (37.5%)
	≤ 40	140 (62.5%)
ALT	> 40	40 (17.9%)
	≤ 40	184 (82.1%)
LDH (U/L)	> 1000	14 (6.7%)
	500–1000	126 (60.0%)
	≤ 500	70 (33.3%)
INR	> 1.5	9 (3.3%)
	≤ 1.5	235 (96.7%)
PTT (sec)	> 40	21 (9.3%)
	≤ 40	204 (90.7%)

*Note:* Data are presented as number (percentage) of patients. All values represent initial laboratory measurements obtained at hospital admission (or within the first 24 h). Pre‐influenza baseline laboratory data were not available for comparison.

### 3.4 Correlations Between Admission Biomarkers

To further characterize the relationships among admission inflammatory and organ injury markers, we calculated pairwise correlation coefficients between ESR, CRP, creatinine (Cr), blood urea nitrogen (BUN), AST, ALT, and LDH. In Pearson correlation analysis, a very strong positive correlation was observed between AST and ALT (r = 0.87), and moderate positive correlations were found between AST and LDH (r = 0.43) and ALT and LDH (r = 0.36), indicating that liver enzymes and LDH tended to increase in parallel. CRP showed weak‐to‐moderate correlations with ESR (r = 0.43) and LDH (r = 0.32), consistent with a shared systemic inflammatory response. Renal markers were closely related (Cr and BUN, r = 0.76), while their correlations with LDH were weaker (r = 0.24–0.26). Similar patterns were observed using Spearman rank correlation coefficients (data not shown), supporting the robustness of these associations. To provide a comprehensive overview of these inter‐relationships, a full correlation matrix with corresponding significance levels is presented in Table 4.

**Table 4:** Correlation matrix of admission biomarkers.

Biomarker	ESR	CRP	Cr	BUN	AST	ALT	LDH
ESR	1.00	0.43^a^	0.18^b^	0.15^b^	0.20^b^	0.14	0.26^a^
CRP	0.43^a^	1.00	0.22^b^	0.19^b^	0.28^a^	0.21^b^	0.32^a^
Cr	0.18^b^	0.22^b^	1.00	0.76^a^	0.20^b^	0.17^b^	0.24^b^
BUN	0.15^b^	0.19^b^	0.76^a^	1.00	0.18^b^	0.15^b^	0.26^a^
AST	0.20^b^	0.28^a^	0.20^b^	0.18^b^	1.00	0.87^a^	0.43^a^
ALT	0.14	0.21^b^	0.17^b^	0.15^b^	0.87^a^	1.00	0.36^a^
LDH	0.26^a^	0.32^a^	0.24^b^	0.26^a^	0.43^a^	0.36^a^	1.00

Abbreviations: ALT, alanine aminotransferase; AST, aspartate aminotransferase; BUN, blood urea nitrogen; CRP, C‐reactive protein; Cr, creatinine; ESR, erythrocyte sedimentation rate; LDH, lactate dehydrogenase.

^a^ *p* < 0.01; *p* < 0.05. All *p*‐values are two‐sided.

^b^ Data are presented as Pearson correlation coefficients (*r*).

Univariate analysis revealed significant associations between prolonged hospitalization (> 5 days) and age ≥ 65 years (*p* = 0.008), female sex (*p* = 0.034), opioid/opium use (*p* = 0.031), cardiovascular disease (*p* = 0.047), and oxygen saturation < 93% (*p* = 0.019). Febrile patients had shorter hospital stays (*p* = 0.035). ICU admission was significantly associated only with oxygen saturation < 93% (*p* = 0.001). Mortality was significantly higher in patients with symptom duration > 3 days (*p* = 0.011), oxygen saturation < 93% (*p* = 0.006), and chronic pulmonary disease (*p* = 0.047) (Tables 5, 6, 7). Table 5 summarizes the univariate logistic regression results for these outcomes.

**Table 5:** Univariate logistic regression analysis of demographic, clinical, and comorbidity factors associated with prolonged hospitalization, ICU admission, and mortality.

Variable	Prolonged hospitalization OR (95% CI)	*p* value*	ICU admission OR (95% CI)	*p* value*	Mortality OR (95% CI)	*p* value*
Age ≥ 65 years	1.88 (1.17–3.04)	0.008	1.68 (0.79–3.57)	0.187	1.52 (0.60–3.84)	0.384
Female sex	1.50 (1.05–2.15)	0.034	1.91 (0.87–4.18)	0.108	0.68 (0.23–1.96)	0.485
Opium/opioid use	1.94 (1.06–3.56)	0.031	2.96 (0.96–9.15)	0.058	1.36 (0.29–6.35)	0.699
Cardiovascular disease	1.78 (1.01–3.14)	0.047	1.21 (0.48–3.03)	0.691	1.00 (0.25–3.96)	0.997
Oxygen saturation < 93%	2.00 (1.12–3.57)	0.019	6.06 (2.09–17.58)	0.001	8.54 (1.93–37.80)	0.005
Fever (yes)	0.53 (0.30–0.94)	0.035	0.46 (0.13–1.57)	0.215	0.31 (0.04–2.33)	0.260
Symptom duration > 3 days	1.37 (0.84–2.25)	0.207	3.00 (0.98–9.19)	0.054	12.92 (0.69–240.69)	0.094
Chronic pulmonary disease	1.63 (0.91–2.92)	0.100	1.45 (0.52–4.03)	0.475	4.00 (1.04–15.33)	0.044

Abbreviations: CI, confidence interval; OR, odds ratio.

* *p*‐values were calculated using the Wald test for each predictor in the univariate logistic regression model.

**Table 6:** Independent predictors of adverse outcomes (multivariate logistic regression).

Outcome	Independent predictor	Odds ratio (OR)	95% CI	*p* value*
Prolonged hospitalization	Opium use	5.40	1.68–17.34	0.004
	Renal complications	9.27	0.98–87.64	0.052
	LDH > 1000 U/L	8.74	1.45–52.67	0.018
ICU admission	LDH > 1000 U/L	46.91	4.96–443.40	0.001
	LDH 500–1000 U/L	5.32	0.65–43.60	0.120
	Opium use	3.33	0.96–11.53	0.057
Mortality	O_2_ saturation < 93%	7.98	1.61–39.52	0.011
	LDH > 1000 U/L	21.66	2.01–233.22	0.011

*Note: p*‐values indicate the statistical significance of each predictor in the multivariate model, testing the null hypothesis that the OR is equal to 1 (no association) after adjusting for other variables.

Abbreviation: CI, confidence interval.

**Table 7:** ROC analysis and diagnostic performance for clinical outcomes.

Outcome	Biomarker	AUC	*p* value*	Cut‐off	Sensitivity (%)	Specificity (%)
Prolonged hospitalization	NLR	0.496	0.942	—	—	—
	LDH	0.680	0.002	539	70.5	55.9
	CRP	0.628	0.027	33.0	70.5	45.9
	ESR	0.659	0.006	23.0	72.7	52.5
ICU admission	NLR	0.526	0.758	—	—	—
	LDH	0.757	0.002	655	71.4	68.6
	CRP	0.779	0.001	88.5	71.4	72.1
	ESR	0.654	0.066	29.5	71.4	53.5
Mortality	NLR	0.506	0.959	—	—	—
	LDH	0.750	0.020	633	87.5	63.3
	CRP	0.747	0.021	88.5	75.0	71.1
	ESR	0.678	0.097	38.5	75.0	68.9

Abbreviations: AUC, area under the curve; CI, confidence interval; ROC, receiver operating characteristic.

* *p*‐values test the null hypothesis that the AUC is 0.5, indicating no discriminative ability. Sensitivity and specificity are reported at the optimal cut‐off point for each biomarker.

### 3.5 Multivariate Logistic Regression Analysis

In the multivariate stepwise logistic regression analysis, several independent predictors were identified for adverse clinical outcomes. For prolonged hospitalization (> 5 days), opium use (OR = 5.40, 95% CI: 1.68–17.34, *p* = 0.004) and LDH levels greater than 1000 U/L (OR = 8.74, 95% CI: 1.45–52.67, *p* = 0.018) were significant independent predictors. Renal complications showed a strong association that approached but did not reach statistical significance (OR = 9.27, 95% CI: 0.98–87.64, *p* = 0.052). Regarding ICU admission, LDH > 1000 U/L was the strongest independent predictor (OR = 46.91, *p* = 0.001), followed by LDH levels between 500 and 1000 U/L (OR = 5.32, *p* = 0.12) and opium use (OR = 3.33, *p* = 0.057). For mortality, oxygen saturation below 93% (OR = 7.98, *p* = 0.011) and LDH > 1000 U/L (OR = 21.66, *p* = 0.011) were independently associated with increased risk of death. Independent predictors identified through multivariate logistic regression analysis for each clinical outcome are summarized in Table 6.

### 3.6 ROC Curve Analysis for Predicting Clinical Outcomes

ROC curve analysis was conducted to assess the diagnostic performance of NLR, LDH, CRP, and ESR in predicting prolonged hospitalization, ICU admission, and mortality. The Area Under the Curve (AUC) reflects the discriminative ability of each marker, with higher values indicating better performance.

### 3.7 Prolonged Hospitalization

LDH (AUC = 0.680; *p* = 0.002), ESR (AUC = 0.659; *p* = 0.006), and CRP (AUC = 0.628; *p* = 0.027) demonstrated statistically significant predictive ability, while NLR did not (AUC = 0.496; *p* = 0.942). Cut‐off values: LDH ≥ 539, CRP ≥ 33.0, ESR ≥ 23.0.

### 3.8 ICU Admission

LDH (AUC = 0.757; *p* = 0.002) and CRP (AUC = 0.779; *p* = 0.001) were strong predictors. ESR showed borderline significance (*p* = 0.066). Cut‐off values: LDH ≥ 655, CRP ≥ 88.5, ESR ≥ 29.5.

### 3.9 Mortality

LDH (AUC = 0.750; *p* = 0.020) and CRP (AUC = 0.747; *p* = 0.021) were significant predictors of mortality. ESR had borderline significance (*p* = 0.097), and NLR showed no predictive value.

The diagnostic performance of inflammatory biomarkers for predicting clinical outcomes is summarized in Table 7.

**Figure 1:**
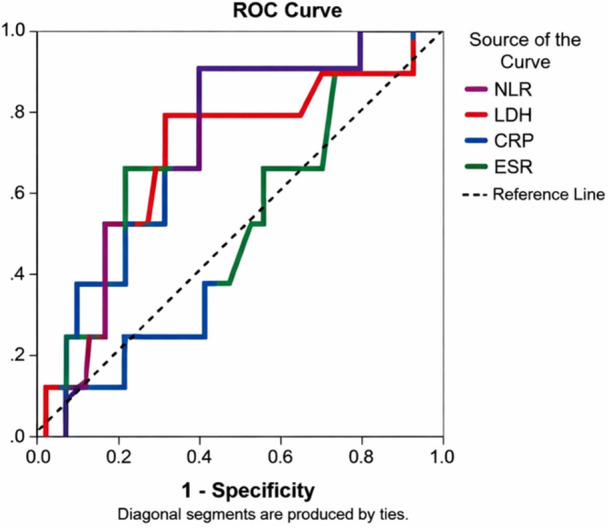
Receiver operating characteristic (ROC) curve analysis for predicting in‑hospital mortality using NLR, LDH, CRP, and ESR. The ROC curves illustrate sensitivity versus 1‑specificity for each biomarker. The diagonal dashed black line represents the reference (chance) line (AUC = 0.5). Among the evaluated markers, LDH (AUC = 0.750) and CRP (AUC = 0.747) demonstrated strong and statistically significant discriminative ability for predicting mortality. ESR showed moderate predictive performance, whereas NLR demonstrated no significant discriminative ability (AUC ≈ 0.5).

Figure 1. displays ROC curves comparing the predictive performance of LDH, CRP, and ESR for mortality. LDH and CRP showed the highest AUC values (0.750 and 0.747, respectively).

Based on the identified ROC cut‑off values and multivariate odds ratios, we constructed a simplified risk stratification framework for ICU admission and in‑hospital mortality (Table 8). This framework is intended as a pragmatic tool to guide hypothesis generation and bedside risk assessment rather than a validated clinical score.

Proposed, non‐validated framework based on current data; requires external validation

**Table 8:** Suggested risk stratification for ICU admission and in‐hospital mortality based on LDH, CRP, and oxygen saturation.

Risk category	Criteria (examples based on study cut‐offs)	Clinical implication
Lower risk	LDH < 655 U/L, CRP < 88.5 mg/L, SpO_2_ ≥ 93%	Standard ward monitoring
Intermediate risk	LDH 655–999 U/L and/or CRP ≥ 88.5 mg/L, SpO_2_ ≥ 93%	Closer monitoring; consider early escalation
Higher risk (ICU/mort.)	LDH ≥ 1000 U/L and/or SpO_2_ < 93%; often with CRP ≥ 88.5 mg/L	Strong consideration for ICU or step‐up care

## 4 Discussion

This study aimed to identify clinical and laboratory predictors of poor outcomes, including prolonged hospitalization, ICU admission, and in‐hospital mortality, among influenza‐positive patients admitted during the 2022–2023 season. Several clinical parameters (e.g., age, oxygen saturation, opium use, cardiovascular disease) and laboratory markers (e.g., CRP, LDH, AST, ALT) were found to be significantly associated with adverse outcomes. Consistent with previous literature, low oxygen saturation (< 93%) emerged as a strong predictor of ICU admission and mortality [13]. Hypoxemia reflects extensive pulmonary involvement, which may signal the need for intensive care or mechanical ventilation [14]. Similarly, elevated LDH and CRP levels were independently associated with all three adverse outcomes. LDH, a marker of tissue injury and cellular stress, and CRP, an acute‐phase reactant, have been widely reported in previous studies as markers of disease severity in respiratory infections [15, 16]. To our knowledge, this is among the first studies conducted in the post‐COVID‐19 era that evaluates a comprehensive set of prognostic outcomes, including prolonged hospitalization, ICU admission, and in‐hospital mortality, in a single influenza‐positive patient cohort using only basic, widely available clinical and paraclinical markers. While prior research has assessed isolated outcomes or relied on advanced clinical scoring systems, our findings demonstrate that standard laboratory indices such as LDH, CRP, and liver enzymes can serve as effective, low‐cost alternatives for early risk stratification, particularly in resource‐limited hospital settings. In line with our findings, multiple studies have highlighted the prognostic role of elevated liver enzymes [17, 18]. Elevated AST and ALT may reflect systemic inflammation or direct hepatic involvement due to viral infection or secondary complications like hypoperfusion or sepsis [19]. Interestingly, opium use was independently associated with both prolonged hospitalization and ICU admission. While this association has been noted in some regional studies, the exact pathophysiological mechanism remains uncertain. Potential explanations include immunosuppression, altered pain perception, delayed healthcare‐seeking behavior, or overlapping comorbidities [20, 21]. While numerous studies have identified age as an independent predictor of ICU admission and mortality in patients with respiratory infections [22, 23], our findings did not reveal a statistically significant association in multivariate analysis. This discrepancy may stem from differences in sample size, admission policies, or population characteristics. Notably, a few studies have also reported similar non‐significant associations, highlighting the potential influence of context‐specific variables [24, 25]. Similarly, although lymphopenia and elevated NLR have been suggested as predictors of severity in respiratory infections like COVID‑19 and influenza, our multivariate analysis aligns with findings from a large cohort study in community‑acquired pneumonia, where NLR lost its prognostic significance for mortality and invasive ventilation after adjusting for clinical severity scores [26]. ROC analysis further reinforced the utility of LDH and CRP as predictive markers, with acceptable AUCs for all outcomes. Notably, LDH ≥ 633 and CRP ≥ 88.5 were strong cutoffs for predicting mortality, aligning with thresholds reported in prior literature [27, 28]. The correlation analysis of admission biomarkers revealed that liver enzymes (AST and ALT) were strongly correlated with each other (*r* = 0.87) and moderately correlated with LDH (AST–LDH: *r* = 0.43; ALT–LDH: *r* = 0.36), suggesting that these markers tend to rise in parallel as indicators of tissue injury. CRP showed weak‐to‐moderate correlations with ESR (*r* = 0.43) and LDH (*r* = 0.32), consistent with a shared systemic inflammatory response. Renal markers (creatinine and BUN) were closely correlated (*r* = 0.76), while their correlations with LDH were weaker (*r* = 0.24–0.26). These interrelationships support the potential combined utility of these biomarkers for risk stratification. Of note, pre‐influenza baseline laboratory data were not available, limiting our ability to distinguish infection‐related changes from pre‐existing conditions. To translate these findings into a more practical clinical framework, we proposed a simplified risk stratification scheme (Table 8) based on the identified cut‑off values for LDH, CRP, and oxygen saturation. In this scheme, moderately elevated LDH and CRP levels (e.g., LDH ≥ 655 U/L and CRP ≥ 88.5 mg/L) identify patients who may benefit from closer monitoring and timely consideration of ICU transfer, whereas very high LDH levels (≥ 1000 U/L) combined with hypoxemia (SpO_2_ < 93%) highlight a subgroup at particularly high risk of in‑hospital death. Although this approach is not intended as a definitive scoring system, it provides a pragmatic, hypothesis‑generating tool that may help clinicians prioritize high‑risk patients, especially in resource‑constrained settings. External validation in prospective, multicenter cohorts is warranted before implementation in routine practice. Despite the relatively large sample size and comprehensive variable assessment, several limitations should be acknowledged. This single‐center study, conducted in a tertiary hospital in northern Iran, may limit the generalizability of the findings. The retrospective design introduces potential selection bias and incomplete data capture, although efforts were made to ensure data accuracy. Virological subtyping was not performed, precluding evaluation of strain‐specific effects on severity and outcomes. National influenza surveillance reports from Iran during the 2022–2023 season indicated that influenza A (H3N2) was the predominant circulating subtype, with lower‐level activity of A(H1N1) pdm09 and minimal influenza B. This epidemiological context may partly explain the relatively severe clinical presentations and elevated inflammatory markers observed in our cohort [29]. Additionally, only hospitalized influenza‐positive patients were included; thus, comparisons with non‐admitted cases and identification of factors associated with hospitalization were not possible. Detailed information on blood pressure control, antihypertensive adherence, and serial measurements during hospitalization was unavailable. The lack of pre‐influenza baseline laboratory data limited our ability to distinguish infection‐related abnormalities from pre‐existing conditions. Influenza vaccination status was unavailable for most patients due to incomplete documentation in the electronic medical records, preventing assessment of the potential protective effect of vaccination against adverse outcomes. Information regarding the timing, dosage, and duration of antiviral therapy (such as oseltamivir) during hospitalization was not systematically recorded. Furthermore, data on secondary bacterial pneumonia or other coinfections were not systematically collected because of inconsistent documentation and the lack of routine microbiological confirmation for all patients, which limited our ability to evaluate their potential contribution to disease severity and outcomes. Prospective multicenter studies with standardized data collection, including vaccination history and systematic screening for secondary bacterial infections, are warranted to address these limitations.

## 5 Conclusion

This study identified several clinical and laboratory parameters, such as low oxygen saturation, elevated CRP, LDH, AST, and ALT levels, as significant predictors of adverse outcomes in hospitalized influenza‐positive patients. These findings highlight the potential utility of readily available biomarkers in early risk stratification and clinical decision‐making. Incorporating such predictors into routine evaluation may help identify high‐risk patients and optimize resource allocation, though further prospective, multicenter studies are needed for validation.

## Author Contributions

**Mohsen Daneshmand:** investigation, funding acquisition, methodology, validation, formal analysis, data curation, project administration. **Arefeh Ebrahimian Shiadeh:** writing – review and editing, writing – original draft, validation. **Farzane Sadeghi:** data curation, validation, project administration. **Hemmat Gholinia:** formal analysis, software, data curation. **Farzin Sadeghi:** conceptualization, investigation, methodology, supervision, data curation, writing – review and editing, project administration. **Masomeh Bayani:** conceptualization, validation, supervision, data curation, project administration.

## Conflicts of Interest

The authors declare no conflicts of interest.

## Transparency Statement

The corresponding author (Dr. Farzin Sadeghi) affirms that this manuscript is an honest, accurate, and transparent account of the study being reported; that no important aspects of the study have been omitted; and that any discrepancies from the study as planned (and, if relevant, registered) have been explained.

## Data Availability

The data that support the findings of this study are available on request from the corresponding author. The data are not publicly available due to privacy or ethical restrictions. The datasets generated and/or analyzed during the current study are not publicly available due to patient confidentiality and ethical restrictions (patient data contain potentially identifying information). However, deidentified data are available from the corresponding author upon reasonable request and with appropriate ethical approval.
